# Embryo growth alteration and oxidative stress responses in germinating *Cucurbita pepo* seeds exposed to cadmium and copper toxicity

**DOI:** 10.1038/s41598-024-58635-1

**Published:** 2024-04-14

**Authors:** Smail Acila, Samir Derouiche, Nora Allioui

**Affiliations:** 1 Department of Biology, Faculty of Nature and Life Sciences, University of El Oued, PO Box 789, 39000 El Oued, Algeria; 2 Laboratory of Biology, Environment and Health, University of El Oued, El Oued, Algeria; 3 Department of Cellular and Molecular Biology, Faculty of Nature and Life Sciences, University of El Oued, El Oued, Algeria; 4 Laboratory of Biodiversity and Application of Biotechnology in the Agricultural Field, University of El Oued, El Oued, Algeria; 5 Department of Ecology and Environmental Engineering, Faculty of Nature and Life Sciences and Earth and Universe Sciences, University of May 8th, 1945, Guelma, Algeria

**Keywords:** Zucchini (*Cucurbita pepo* L.), Heavy metals, Embryonic axes, Oxidative stress, Germination, Antioxidants, Plant sciences, Environmental sciences

## Abstract

This study investigated the influence of cadmium (Cd) and copper (Cu) heavy metals on germination, metabolism, and growth of zucchini seedlings (*Cucurbita pepo* L.). Zucchini seeds were subjected to two concentrations (100 and 200 μM) of CdCl_2_ and CuCl_2_. Germination parameters, biochemical and phytochemical attributes of embryonic axes were assessed. Results revealed that germination rate remained unaffected by heavy metals (Cd, Cu). However, seed vigor index (SVI) notably decreased under Cd and Cu exposure. Embryonic axis length and dry weight exhibited significant reductions, with variations depending on the type of metal used. Malondialdehyde and H_2_O_2_ content, as well as catalase activity, did not show a significant increase at the tested Cd and Cu concentrations. Superoxide dismutase activity decreased in embryonic axis tissues. Glutathione *S*-transferase activity significantly rose with 200 μM CdCl_2_, while glutathione content declined with increasing Cd and Cu concentrations. Total phenol content and antioxidant activity increased at 200 μM CuCl_2_. In conclusion, Cd and Cu heavy metals impede zucchini seed germination efficiency and trigger metabolic shifts in embryonic tissue cells. Response to metal stress is metal-specific and concentration-dependent. These findings contribute to understanding the intricate interactions between heavy metals and plant physiology, aiding strategies for mitigating their detrimental effects on plants.

## Introduction

For several decades, the increase in human activities such as industrial waste, urban transport, and excessive use of fertilizers in the agricultural sector has been the cause of environmental pollution by heavy metals^1^. The accumulation of heavy metals in ecosystems has led to the deterioration of environmental quality, the decline of forests, and the reduction of agricultural productivity over the years^2^.

In plants, certain metals are often essential for the development of biological processes, and these are known as trace elements or micronutrients (e.g., iron, zinc, copper, etc.), but many of them can prove to be contaminants for various forms of life when their concentration exceeds a certain threshold. Even at low concentrations, heavy metals such as cadmium (Cd) and lead (Pb) can be harmful contaminants^3,4^. Plants exposed to heavy metal stress suffer from growth retardation, reduced yield, increased accumulation of reactive oxygen species, and altered antioxidant metabolism, especially the enzymatic system. This stress can also cause alteration of the structure and permeability of the cell membrane, imbalance of endogenous hormones, changes in nutrient absorption and distribution. Additionally, it damages the ultrastructure of functional organelles and inevitably impairs photosynthesis and respiratory capacity^4,5^.

Previous studies have provided limited information on how plants respond to equivalent amounts of cadmium or copper exposure. However, the available research suggests that plants are more vulnerable to copper compared to cadmium^6^. Furthermore, the results of another study confirmed that the effects of cadmium and copper on certain metabolic processes were variable between the parameters of germination^7^. However, the inhibition of this process appears to rely on various factors, such as the type and concentration of metal, the duration of seed exposure, the specific plant species and variety, and even the nature of the seed coat, as noted by Sethy and Ghosh^8^ and Seneviratne, et al.^9^. This inhibition is often associated with disruptions in respiratory metabolism, according to Smiri, Chaoui and El Ferjani^10^ and Chugh and Sawhney^11^.

It is certain that heavy metal ions such as Cd^2+^ and Cu^2+^ have toxic effects on seed germination by altering the structure of certain proteins, inhibiting their enzymatic activity^12^, inducing oxidative stress, and disrupting the processes of cellular oxidation–reduction metabolism of seedling tissues^13,14^. ROS, which include hydrogen peroxide, superoxide radicals, singlet oxygen, and hydroxyl radicals, are found in all plant cells because they are constantly generated as undesired byproducts during various metabolic processes. These metabolic pathways primarily occur in the chloroplasts, mitochondria, and nitrogen-fixing nodules^15,16^. Every type of ROS plays a role in the signaling pathways and responses to environmental factors^17^.

Faced with stressful situations, plants mobilize antioxidant defense systems, which are of an enzymatic nature (catalase, superoxide dismutase, peroxidase, glutathione reductase, glutathione *S*-transferase, glutathione peroxidase, etc.) or non-enzymatic nature (Vitamins C and E, glutathione, phenols, etc.), involving different compounds that have an affinity for free radicals^18,19^. Nevertheless, antioxidant enzymes are responsible for regulating and decreasing the concentrations of ROS^16^. Superoxide dismutase (SOD) converts superoxide to hydrogen peroxide (H_2_O_2_) and oxygen (O_2_), while ascorbate peroxidase (APX), guaiacol peroxidase (GPX), catalase (CAT), and glutathione reductase (GR) convert H_2_O_2_ to water (H_2_O) and O_2_^15,16^.

Germination and early seedling growth are the most sensitive physiological stages of a plant, and they are regulated by several hormonal interactions, as well as environmental factors^20^. In addition, these stages are more sensitive to metal pollution^21^. The effect of heavy metals on crop plants differs from one species to another, and *Cucurbita pepo* species has been used as a biological model by several authors^22–24^, to test the toxicity of metallic elements in the early stages of plant development.

Zucchini (*Cucurbita pepo* L.) belongs to the Cucurbitaceae; it is grown all over the world for food supply^25^. Its economic and medical importance is increasingly apparent for its nutrients and richness in bioactive compounds, including phenols, flavonoids, vitamins, amino acids, carbohydrates, and minerals^26,27^. In addition, it is characterized by high protein content^28^.

The objective of this work is to study the effect of cadmium (Cd) and copper (Cu) on the germination and growth of zucchini (*C. pepo*) seedlings and to evaluate some biochemical and phytochemical responses of the embryonic axes under the effect of metallic stress (Cd, Cu) in the medium.

## Material and methods

### Seed preparation

In the laboratory, zucchini seeds (*Cucurbita pepo* L.), Quarantaine variety, were thoroughly washed with tap water to eliminate residues of the active ingredient (thiram) used for seed treatment. Subsequently, they were disinfected by soaking in a 10% solution of sodium hypochlorite (NaClO, 12°) for 10 min, followed by abundant rinsing with distilled water to remove any traces of NaClO. The initial state of the seeds (non-germinated) was achieved by soaking them in distilled water at 5°C for 30 min^29^.

### Germination test and metallic treatments

After 30 min of soaking in distilled water, zucchini *C. pepo* seeds, taken at random, were germinated in plastic dish (15 cm in diameter × 2 cm high), sterilized and lined with filter paper, at a rate of 50 seeds/dish. The used metals were cadmium and copper in the form of salts: cadmium chloride (CdCl_2_) and copper chloride (CuCl_2_). Zucchini seeds were moistened in distilled water (for the control) or treated with metallic solutions at two concentrations, 100 and 200 μM (15 to 20 mL/dish/day), and 3 replicates were carried out for each treatment. The germination test was performed in an incubator, under average temperature conditions of 27 ± 1 °C and in total darkness for 8 days.

The selection of metal treatment concentrations was based on preliminary experiments. The highest concentration of 200 µM of CdCl_2_ and CuCl_2_ was chosen because this concentration resulted in more than 50% inhibition of zucchini seedling growth parameter (data not shown).

### Studied parameters and performed measurements

#### Germination criteria

- The average germination rate (GP) is expressed as the maximum percentage of germinated seeds after 8 days out of the total number of sown seeds^30^. A seed is considered to have germinated when its radicle is at least 2 mm in length^31^.
- The seed vigor index (SVI,) is determined after 8 days of sowing according to the formula described by Abdul‐Baki and Anderson^32^: SVI = (GP × EAL)/100, where, EAL: total length of the embryonic axes (radicle + hypocotyl), and GP: average germination rate.

### Lengths and dry weights of embryonic axes of *C. pepo* seedlings

For eight (8) day old zucchini seedlings, the length of the embryonic axes EAL (cm) was measured for 20 samples of each treatment, using a millimeter paper. The dry weight of the embryonic axes EAW (mg) was determined, after drying the samples at 70°C for 48 h, using an analytical balance.

Heavy metal phytotoxicity rate on the growth of the embryonic axes was calculated according to the formulas below described by Wierzbicka, Bemowska-Kałabun and Gworek^33^:

$$EAL\, Phytotoxicity \, \left( \% \right) = \, \left( {\left( {{\text{EAL}}_{{{\text{control}}}} {-}{\text{ EAL}}_{{{\text{treatment}}}} } \right) \, /{\text{ EAL}}_{{{\text{control}}}} } \right) \times {1}00$$

$$EAW \, Phytotoxicity \, \left( \% \right) = \, \left( {\left( {{\text{EAW}}_{{{\text{control}}}} {-}{\text{ EAW}}_{{{\text{treatment}}}} } \right) \, /{\text{ EAW}}_{{{\text{control}}}} } \right) \times {1}00$$

### Biochemical parameters and oxidative stress markers in the embryonic axes of *C. pepo* seedlings

#### Tissue homogenate preparation

The enzymatic extract of the embryonic axes’ tissues (hypocotyl + radicle) of young zucchini seedlings was obtained by the method of Loggini, et al.^34^. Briefly, fresh samples (1 g) were ground in a mortar, with 5 mL of phosphate buffer, after centrifugation at 4°C for 15 min at 12.000 g, the obtained supernatant was used as an extract for the determination of the various enzymatic activities.

### Measurement of hydrogen peroxide (H_2_O_2_) levels

The concentration of hydrogen peroxide (H_2_O_2_) was measured in the embryonic axes of young zucchini seedlings using the method of Velikova, Yordanov and Edreva^35^. This method is based on the absorption spectra of the reaction between hydrogen peroxide (H_2_O_2_) and potassium iodide (KI 1 M) in 0.1% trichloroacetic acid (TCA) at pH 7. The absorbance was measured at 390 nm using a Jenway 6705 spectrophotometer, and the H_2_O_2_ content was expressed in µmole g^-1^ FM.

### Measurement of malondialdehyde (MDA) concentration

Lipid peroxidation was measured according to the method described by Draper and Hadley^36^. Thiobarbituric acid 0.37% (w/v) was added to the homogenate previously precipitated with 20% trichloroacetic acid (w/v). Then the mixture was centrifuged, and the supernatant was heated (100 °C) for 15 min in a boiling water bath. After cooling by a cold-water bath, the absorbance was measured at 532 nm. The concentration of thiobarbituric acid reactive substances (TBARS) was determined using the molecular extinction coefficient of MDA (ε = 1.53 × 10^5^ M^-1^ cm^-1^).

### Determination of catalase (CAT) activity

The activity of catalase (CAT) was assessed using the method of Regoli and Principato^37^. Briefly in test tubes, mix 1 mL of phosphate buffer (0.1 M, pH7.2), 0.975 mL of freshly prepared H_2_O_2_ (0.091 M) and 0.025 mL of the enzyme source (homogenate) which involves determining the speed of decomposition of H_2_O_2_ by measuring the decrease in colorimetric absorbance at 240 nm over one minute. The activity of catalase was expressed in IU/g protein using a molar extinction coefficient (ε) of 39.4 mM^−1^ cm^−1^.

### Determination of superoxide dismutase (SOD) activity

The activity of superoxide dismutase (SOD) was analyzed using the nitroblue tetrazolium (NBT) method of Beauchamp and Fridovich^38^. In brief, 1 mL of EDTA-Met was added to 1.95 mL of TBS (pH 7.6) and 0.05 mL of homogenate. The mixture is mixed with 0.085 mL of NBT (75 µM) and 0.022 mL riboflavin (2 µM). This reaction has a strong absorbance at 560 nm. One unit (U) of SOD is defined as an amount of protein that inhibits the reduction of NBT by 50%. The estimated activity of SOD was expressed in IU/g of protein.

### Determination of glutathione-*S*-transferase (GST) activity

Glutathione-*S*-transferase (GST) activity of tissues was measured spectrophotometrically by the method of Habig, Pabst and Jakoby^39^. Briefly, 0.83 mL of phosphate buffer and 0.05 mL of CDNB (1-chloro 2, 4 dinitrobenzene) as electrophilic substrate was added to 0.1 mL of GSH in the presence of 0.02 mL of enzyme source (homogenate) and forms a colored GSH-substrate complex, detected at 340 nm. The activity of GST was expressed in terms of nmol CDNB/GSH conjugate formed/min/mg protein.

### Measurement of reduced glutathione (GSH) levels

GSH concentration was performed with the method described by Weckbecker and Cory^40^ based on the development of a yellow color when DTNB (5,5′-dithio-bis-2-nitro benzoic acid) is added to compounds containing sulfhydryl groups. In brief, 0.8 mL of homogenate was added to 0.2 mL of 0.25% sulphosalylic acid and tubes were centrifuged at 2500*g* for 15 min. Supernatant (0.5 mL) was mixed with 0.025 mL of 0.01 M DTNB and 1 mL of buffer solution of TBS (pH 7.4). Finally, absorbance at 412 nm was recorded. Total GSH content was expressed as µmol/mg protein.

### Phytochemical analysis of embryonic axes of *C. pepo* seedlings

#### Extraction of embryonic axis tissues

Embryonic tissue samples of young zucchini seedlings (1 g of dry matter) were weighed, and 20 mL of concentrated methanol was added to hermetic flasks. After agitation, the mixtures were placed in the dark at room temperature for 48 h. Subsequently, the mixtures were paper-filtered under a vacuum suction system. The obtained filtrate was kept at ambient temperature and protected from light for 48 h. The resulting dry residues were then stored in the refrigerator at 4 °C until measurement.

#### Total polyphenol content (TPC) assay

The determination of the total polyphenol content was carried out according to the Folin-Ciocalteu method in an alkaline medium^41^. A quantity of 100 µL of the extract (1 mg/mL) was mixed with 50 µL of the freshly prepared Folin-Ciocalteu reagent (3%), and 1 mL of 7.5% sodium bicarbonate (Na_2_CO_3_) was added. The mixture was incubated at room temperature for 30 min, and the absorbance of the resulting blue color was measured at λmax = 760 nm using a Shimadzu UV–VIS spectrophotometer. The quantification was performed using the standard curve of gallic acid, and the results were expressed in micrograms of gallic acid equivalents (GAE) per milligram of dry matter (µg GAE/mg DM).

#### Total flavonoid content (TFC) assay

The flavonoid content of the extracts was determined using the aluminum chloride (AlCl_3_) colorimetric method described by Dewanto et al.^42^, with some modifications. A quantity of 750 µL of the extract (1 mg/mL) (prepared in methanol) was added to 750 µL of the AlCl_3_ solution (2% dissolved in methanol). After ten minutes, the absorbance was measured against the prepared reagent blank at λmax = 415 nm. Flavonoid concentrations were calculated based on the calibration curve established with quercetin. The results were expressed as micrograms of quercetin equivalents (QE) per gram of dry matter (µg QE/g DM).

#### Antioxidant activity assay (DPPH test)

To investigate the antioxidant activity of different extracts of the embryonic axes of *C. pepo* seedlings, the colorimetric method based on DPPH (1,1-diphenyl-2-picrylhydrazyl) was employed, as described by Blois^43^. A volume of 200 μL of the extract was added to 900 μL of a freshly prepared methanolic solution of DPPH (0.04%). For the negative control, 200 μL of methanol was mixed with 900 μL of a methanolic solution of DPPH at the same concentration. The mixture was then incubated in the dark at room temperature for 30 min. Subsequently, the absorbance was measured at 517 nm using a UV–visible spectrophotometer. The radical scavenging percentage *I*(%) was calculated using the following equation: *I*(%) = [(A0 − Ai)/A0] × 100, where A0 represents the absorbance of the control (DPPH solution without extract), and Ai represents the absorbance in the presence of the extract.

### Statistical analysis

Results are expressed as the mean ± standard error (SE), and data comparisons were made by Fisher's test (LSD), for p < 0.05, using Minitab data analysis software (version 16.0).

### Ethical approval

The plant material used and all the steps of experimentation on *C. pepo* var. Quarantaine seeds are in compliance with relevant institutional, national, and international guidelines and legislation.

## Results

### The germination rate of zucchini seeds (*C. pepo* L.)

**Figure 1:**
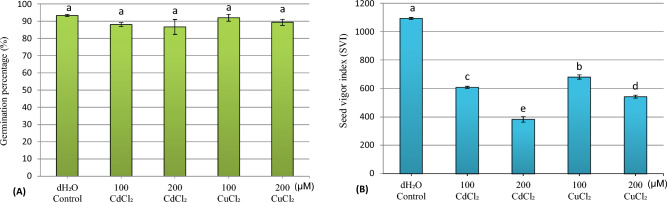
Average seed germination rate (GP) (**A**) and seed vigor index (SVI) (**B**) of zucchini seeds (*Cucurbita pepo* L.) after 8 days of germination as a function of the concentrations of CdCl_2_ and CuCl_2_. *dH*_*2*_*O* distilled water. Data are presented as means ± SE (n = 3 independent experiments). Means not sharing the same letter are significantly different at p ≤ 0.05 by Fisher's LSD test.

The results obtained after 8 days of germination (Fig. 1A) revealed a non-significant reduction (p = 0.313) in the average germination rates of zucchini seeds when exposed to different concentrations of metal solutions (CuCl_2_ and CdCl_2_). The control seeds exhibited a germination rate of 93.33%. Interestingly, the presence of 200 µM cadmium salt (CdCl_2_) led to a germination rate of 86.67%, which did not significantly differ from the control. Moreover, at a concentration of 100 µM copper chloride (CuCl_2_), the decrease in germination was minimal, just 1.43%, maintaining a high germination rate of 92%.

### Seed vigor index (SVI)

The obtained results (Fig. 1B) show a significant decrease (P = 0.001) in the seed vigor index (SVI) under the influence of the used concentrations of cadmium chloride (CdCl_2_) and copper chloride (CuCl_2_). The highest SVI (1092.47) was recorded in the control seeds, while the minimum value of this index (382.6) was noted at the concentration of 200 µM CdCl_2_, with a decrease of the order of 64.98%. At a concentration of 100 µM CuCl_2_, where the SVI value was approximately 680.8, a reduction rate of 37.68% was observed.

### Length (EAL) and dry biomass (EAW) of embryonic axes of zucchini seedlings (*C. pepo* L.)

The results (Table 1) show a very highly significant decrease (p = 0.000) in the average length and average dry weight of the embryonic axes of zucchini seedlings under the influence of metal-induced stress in the medium.

**Table 1:** Effects of heavy metals (Cu, Cd) on the total length (EAL), dry weight (EAW), and phytotoxicity rate (%) of zucchini seedling embryonic axes (*Cucurbita pepo* L.) after 8 days of germination.

Metal solution	Treatment	EAL (cm)	EAL phytotoxicity (%)	EAW (mg)	EAW phytotoxicity (%)
Control	dH_2_O	11.71 ± 0.31^a^	–	22.79 ± 1.05^a^	–
CdCl_2_	100 µM	6.90 ± 0.33^bc^	43.98 ± 3.77^bc^	17.32 ± 0.53^b^	26.47 ± 3.64^b^
	200 µM	4.42 ± 0.27^d^	63.02 ± 2.61^a^	16.32 ± 0.62^bc^	31.36 ± 4.09^ab^
CuCl_2_	100 µM	7.40 ± 0.40^b^	38.93 ± 4.25^c^	15.63 ± 0.67^bc^	33.72 ± 3.80^ab^
	200 µM	6.07 ± 0.21^c^	49.63 ± 2.54^b^	14.20 ± 1.02^c^	40.29 ± 5.10^a^
P. value, and significance level		0.000***	0.000***	0.000***	0.141^NS^

Data are presented as the means ± SE (n = 20). Values in the same column not sharing the same letter are significantly different at p ≤ 0.05 by Fisher's LSD test. ***Extremely significant, ^NS^not significant.

*dH*_*2*_*O* distilled water.

The embryonic axis length (EAL) of 11.71 cm was noted in the control (untreated seedlings), while at the concentration of 200 µM of CdCl_2_, the EAL was 4.42 cm, indicating a maximum phytotoxicity of 63.02% (Table 1). At 100 µM of CuCl_2_, the EAL was 7.4 cm, with a minimal toxicity of 38.93%.

The embryonic axis dry weight (EAW) of 22.79 mg was recorded in zucchini seedlings treated with distilled water (control), while at 200 µM of CuCl_2_, the EAW was 14.2 mg, indicating a maximum phytotoxicity of 40.29% (Table 1). At 100 µM of CdCl_2_, the EAW was 17.32 mg, showing the lowest toxic effect of 26.47%.

### Biochemical parameters of the embryonic axes of *C. pepo* seedlings

#### Hydrogen peroxide and malondialdehyde content

**Figure 2:**
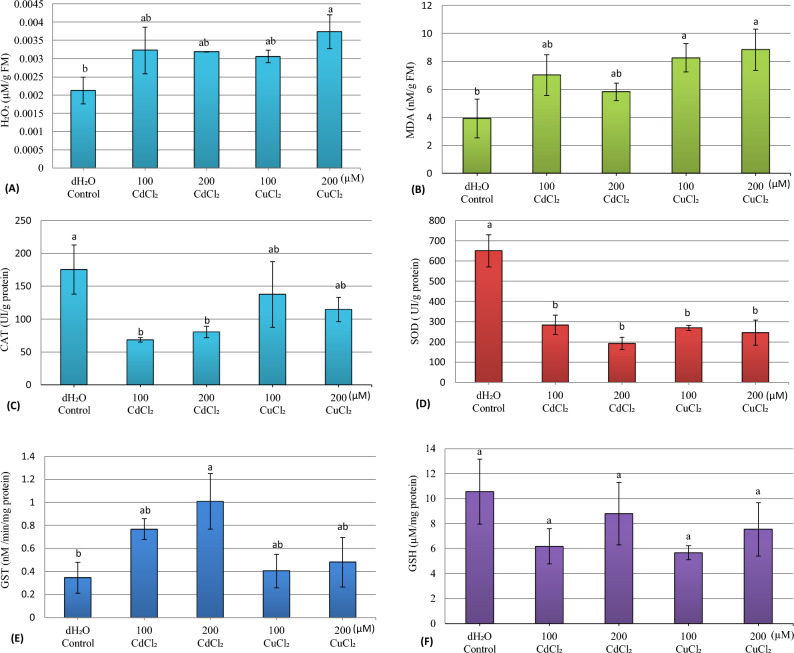
Hydrogen peroxide (H_2_O_2_) levels (**A**), malondialdehyde (MDA) content (**B**), catalase activity (CAT) (**C**), superoxide dismutase activity (SOD) (**D**), glutathione *S*-transferase activity (GST) (**E**), and glutathione content (GSH) (**F**) of zucchini seedling embryonic axes (*Cucurbita pepo* L.) after 8 days of germination as a function of CdCl_2_ and CuCl_2_ concentrations. *dH*_*2*_*O* distilled water. Data are presented as means ± SE (n = 3). Means not sharing the same letter are significantly different at p ≤ 0.05 by Fisher's LSD test.

The results (Fig. 2A, B) show that for the embryonic axes tissues of young zucchini seedlings, the applied concentrations of cadmium chloride and copper chloride lead to a non-significant increase (P = 0.144 and P = 0.107) in the hydrogen peroxide and malondialdehyde content, respectively, compared to control seedlings. The highest value of H_2_O_2_ content (0.0037 µM/g FM) and the highest value of MDA content (8.83 nM/g FM) were noted at 200 µM of CuCl_2_.

#### Enzymatic and non-enzymatic antioxidants content

The obtained results (Fig. 2C) show that the measurement of the enzymatic activity of catalase of the embryonic axes tissues of the zucchini seedlings (*C*. *pepo* L.) revealed a non-significant decrease (p = 0.147) under the effect of metallic stress. The highest value of CAT activity (137.4 IU/g protein) was observed at the concentration of 100 µM CuCl_2_, while the lowest value (68.39 IU/g protein) was recorded at 100 μM of CdCl_2_, against 175.3 IU/g proteins in the control.

On the other hand, the measured superoxide dismutase activity for the embryonic tissues of zucchini seedlings, subjected to metallic stresses (Fig. 2D) exhibited a highly significant decrease (p = 0.002) compared to the control seedlings and the lowest value (192.7 IU/g protein) was noted at 200 µM of CdCl_2_. On the other hand, the glutathione *S*-transferase activity of the embryonic tissues (Fig. 2E) showed a non-significant increase (p = 0.209) under the effect of metal stresses, compared to the control. At 200 µM of CdCl_2_, the GST activity was recorded as 1.008 nM/min/mg protein, while at 100 μM of CuCl_2_, the GST activity was 0.404 nM/min/mg protein, compared to 0.346 nM/min/mg protein in the control seedlings.

For the glutathione (GSH) content of the embryonic axes of young zucchini seedlings (Fig. 2F), the results revealed no significant decrease (P = 0.452) under the effect of CdCl_2_ and CuCl_2_ salts compared to the control seedlings. The minimum GSH content (5.66 µM/mg protein) was recorded at 100 µM CuCl_2_.

#### Total polyphenol content (TPC), total flavonoid content (TFC) and antioxidant capacity (*I *(%)) of the embryonic axes of *C. pepo* seedlings

The results presented in Table 2 demonstrate a highly significant effect (p = 0.000) of heavy metal stress on the total phenolic and total flavonoid contents of embryonic axis tissues.

**Table 2:** Total phenol content (TPC), total flavonoid content (TFC), and antioxidant activity (*I* (%)) of zucchini seedling embryonic axes (*Cucurbita pepo* L.) exposed to different concentrations of heavy metal salts (CdCl_2_ and CuCl_2_) after 8 days of germination.

Metal solution	Treatment (µM)	TPC (µg GAE mg−^1^ DW)	TFC (µg QE mg−^1^ DW)	DPPH *I *(%)
Control	dH_2_O	10.87 ± 0.76^c^	1.05 ± 0.44^c^	45.23 ± 5.37^bc^
CdCl_2_	100 (µM)	38.49 ± 5.95^b^	1.85 ± 1.39^c^	49.39 ± 4.45^abc^
	200 (µM)	5.54 ± 0.94^c^	2.88 ± 0.07^c^	38.28 ± 6.47^c^
CuCl_2_	100 (µM)	10.34 ± 2.24^c^	10.72 ± 1.39^b^	58.74 ± 8.53^ab^
	200 (µM)	70.02 ± 0.24^a^	19.66 ± 0.38^a^	64.44 ± 2.82^a^
P. value, and significance level		0.000***	0.000***	0.044*

Data are presented as the means ± SE (n = 3). Values in the same column not sharing the same letter are significantly different at p ≤ 0.05 by Fisher's LSD test. ***Extremely significant, *significant.

*dH*_*2*_*O* distilled water.

For TPC content in the embryonic axes, we recorded a variable effect depending on the nature and the concentration of the metal to which the seedlings were subjected. For CdCl_2_, an increase in TPC at the concentration of 100 µM (38.49 µg GAE mg^−1^ DW) and a decrease (5.54 µg GAE mg^−1^ DW) at 200 µM were noted, compared to the control seedlings (10.87 µg GAE mg^−1^ DW). For CuCl_2_, an average value that was non-significantly different from the control (10.34 µg GAE mg^−1^ DW) was recorded at the concentration of 100 µM, while a remarkable increase in TPC was obtained at 200 µM (70.02 µg GAE mg^−1^ DW).

The flavonoid content of seedlings subjected to metal stress exhibited a proportional increase, depending on the concentration of cadmium chloride (CdCl_2_) and copper chloride (CuCl_2_) used. The highest values were obtained for CuCl_2_ with a content of 19.66 µg QE mg^−1^ DM at a concentration of 200 µM and 10.72 µg QE mg^−1^ DM at a concentration of 100 µM, against 1.05 µg QE mg^−1^ DM in control seedlings. Seedlings subjected to CdCl_2_ recorded lower flavonoid contents compared to those subjected to CuCl_2_ at 100 µM and 200µM (1.85 µg QE mg^−1^ DM and 2.88 µg QE mg^−1^ DM, respectively).

The results of the DPPH free radical inhibition rates (*I*(%)) of the embryonic tissue extracts (Table 2) show overall that this antioxidant activity increases significantly under the effect of metallic stresses. This is particularly so for CuCl_2_ where a mean maximum inhibition rate (64.44%) was noted at a concentration of 200 µM. However, a lower inhibition rate (38.28%) than that of the control seedlings was recorded in the zucchini seedlings exposed to a concentration of 200 µM of CdCl_2_.

## Discussion

Our results indicate that the presence of cadmium chloride (CdCl_2_) and copper chloride (CuCl_2_) at the tested concentrations (100 and 200 µM) did not have a statistically significant impact on the germination rate of zucchini seeds. The initial phases of germination, marked by the emergence of a 2 mm radicle, proceeded as expected, consistent with previous findings^31^. Interestingly, this process remained active even in germination media treated with metal solutions (Cd and Cu). Certain components of the seed coat's cell walls, including lignin, cellulose, hemicellulose, and pectin, acted as barriers, impeding the movement of metal ions, and sequestering them at the cell wall level^44,45^. This phenomenon persisted at least during the initial stages of germination.

Previous studies by Wierzbicka and Obidzińska^46^ confirmed the impermeability of seed coats to heavy metals, such as lead (Pb), during imbibition in various plant species. Similarly, Kalai, et al.^47^ observed impermeability in barley seeds, and Mihoub, Chaoui and El Ferjani^7^ in pea seeds. These findings suggest that the inhibition of seed germination after exposure to Cd or Cu is not primarily due to reduced water uptake by seed tissues but may involve potential failures in the reserve mobilization process from the endosperm or cotyledons, as well as disruptions in the release of amino acids and transport of soluble sugars.

We observed a significant decrease in the zucchini seed vigor index (SVI) with increasing metal stress in the growth medium. Similar trends were reported by Di Salvatore, Carafa and Carratù^48^ across various plant species (radish, broccoli, lettuce, and tomato) exposed to concentrations of 1024 µM of Cd, Cu, Pb, and Ni. Additionally, Tamas, et al.^49^ found that a concentration of 2 mM AlCl_3_ did not impact the germination of barley seeds but resulted in a notable decrease in radicle growth.

The decline in SVI under environmental metal stress can be primarily attributed to the phytotoxic effects of metal ions (Cd^2+^ and Cu^2+^) on the enzymatic activity of amylases, proteases, and ribonucleases^8^. These effects lead to the inhibition of seed reserve degradation, disrupting various biological processes involved in germination and seedling growth^9^.

Our findings reveal that even at low concentrations (100 µM) of cadmium or copper, the lengths and weights of zucchini seedlings experience a significant reduction compared to the control (Table 1). As supported by Asadi Aghbolaghi, et al.^23^, higher concentrations of cadmium (200 mg/L) can lead to the decline of protein and lipid structures due to the influence of free radicals, resulting in a reduction of these reserves. Additionally, previous studies have shown that oxidative stress induced by osmotic stress^50^ and the involvement of reactive oxygen species (ROS) play a role in programmed cell death during germination and seedling development. The interactions among H_2_O_2_, GA, and ABA hormones in the alveolar layer have also been documented^51^. Furthermore, reduced concentrations of gibberellic acid and the synthesis of alpha- and beta-amylase hydrolyzing enzymes are believed to contribute to a decline in the mobilization of seed reserves under oxidative stress^52^.

The toxic effects of heavy metals on seed metabolic efficiency significantly impact the kinetics of reserve degradation, leading to the depletion of endospermic nutrients, such as soluble sugars and amino acids, vital for seedling development^7,53^. The high toxicity of Cd^2+^ and Cu^2+^ metal ions within plant tissues is attributed to the generation of oxidizing radicals, hindering cell division and elongation, ultimately reducing the growth of young seedlings^54,55^.

Furthermore, the phytotoxic impacts of heavy metals (Cd, Cu) on the growth of zucchini seedlings are consistent with studies conducted by Munzuroglu and Geckil^56^ and Labidi, et al.^57^. These investigations have shown that the dry weights of the embryonic axes, consisting of the hypocotyl and radicle, are notably more affected by copper salt (CuCl_2_) than by cadmium salt (CdCl_2_). Interestingly, the elongation of young zucchini seedlings appears more susceptible to cadmium stress. The detrimental influence of accumulating Cd^2+^ ions become particularly evident in the developing embryonic tissues, leading to a phytotoxic impact, particularly at the cellular level of the radicle, and impairing its growth, consistent with previous findings, as illustrated by Kalai, et al.^47^ and Rucińska-Sobkowiak^58^.

The phytotoxic effects of cadmium (Cd) and copper (Cu) on seed germination and seedling development have been documented by various researchers in different plant species, including zucchini (*C. pepo*)^57^, Atriplex (*A. halimus*)^59^, mung bean (*P. aureus* Roxb)^60^, and rice (*O. sativa*)^61^. Recent research by Asadi Aghbolaghi, et al.^23^ concluded that *C. pepo* seeds exhibit relative tolerance to the toxic effects of cadmium at an early stage of germination, accompanied by a depletion in protein and lipid reserves, as well as a reduction in seed vigor and the activity of antioxidant enzymes such as catalase (CAT), superoxide dismutase (SOD), and peroxidase (POX) in germinated zucchini seeds.

The analysis of oxidative stress parameters revealed a noteworthy escalation in lipid peroxidation activity, as evidenced by heightened levels of H_2_O_2_ and malondialdehyde content. This elevation was accompanied by the initiation of oxidative processes in the embryonic tissues of zucchini seedlings subjected to metal treatments involving cadmium and copper (Cd, Cu). Notably, while these findings corroborate the outcomes presented by Labidi, et al.^57^ in their study on the same species (*C. pepo* L.), as well as the research conducted by Jaouani, et al.^62^ on peas, certain distinctions have emerged. Specifically, when considering the effects of copper treatment (CuCl_2_) at concentrations of 100 µM and 200 µM, a significant difference in H_2_O_2_ levels was observed, accompanied by an analogous trend in MDA content. In contrast, neither concentration of cadmium treatment (CdCl_2_) led to discernible oxidative stress, with no statistically significant difference observed between CdCl_2_ and the control conditions. This disparity in the impact of cadmium compared to copper prompts a closer examination.

Therefore, the ROS formed and accumulated during heavy metal-induced stress react with polyunsaturated fatty acids, resulting in the formation of lipid radicals and reactive aldehydes. These compounds, in turn, lead to a decrease in membrane fluidity and the alteration of membrane proteins^63^. It has been demonstrated that metal stress triggers the production of MDA, which is accompanied by an increase in the content of polyunsaturated fatty acids due to the degradation of cell membranes, which are the primary targets of heavy metal damage^64–66^. On the other hand, the increase in MDA content can be attributed to the inefficiency of the antioxidant system and an imbalance between oxidants and antioxidants in favor of ROS, thus contributing to oxidative stress^67^.

However, an analysis of the results pertaining to the variation of enzymatic and non-enzymatic antioxidants revealed a notable decrease in CAT activity within the zucchini seedling tissues upon exposure to heavy metals, namely cadmium (Cd) and copper (Cu), in the growth medium. Furthermore, this decline in catalase activity was particularly evident in the embryonic axes, and this reduction was found to correlate with elevated concentrations of H_2_O_2_, as noted in the study by Moussa^68^. These observations find resonance in the research conducted by Labidi, et al.^57^ on zucchini and by Sandalio, et al.^69^ on peas, both of which reported comparable findings. Likewise, the findings align with the study conducted by Li, et al.^70^, wherein a reduction in CAT activity in the leaves of two plant species subjected to various Cd treatments was observed. Del Río et al.^71^ provide insight into the catalase activity recorded in the embryonic axes of unstressed zucchini seedlings (control), attributing it to the concentration of the enzyme within the peroxisome and its role in eliminating hydrogen peroxide produced as a result of glycolate oxidase activity. Alternatively, the decrease in CAT activity within the embryonic axes tissues due to the influence of metallic treatments with Cd and Cu can be interpreted either as a consequence of enzyme inhibition by the metallic elements or as a result of reactive oxygen species (ROS) elimination within the zucchini seedling tissues^68^. This decline in CAT activity could be attributed to the accumulation of H_2_O_2_ during the initial phase of the antioxidant defense response, potentially hindering the function of catalase and ultimately leading to a reduction in its activity^72,73^.

Our findings reveal a parallel pattern in the variations of SOD and CAT activities. The decline in SOD activity within zucchini seedlings when subjected to metal stress, specifically cadmium (Cd) and copper (Cu), aligns with the observations made by Labidi, et al.^57^ in their study on zucchini (*Cucurbita pepo* L.), Sandalio, et al.^69^ in their research on peas (*Pisum sativum* L.), and Fatima and Ahmad^74^ in their investigation of onions (*Allium cepa* L.). However, it's worth noting that Mobin and Khan^75^ reported an opposing trend, observing increased SOD activity levels in mustard plants (*Brassica juncea* L.) exposed to cadmium stress. Similarly, Hu, et al.^76^ documented a substantial enhancement in SOD activity within copper-treated wheat seeds (*Triticum aestivum* L.).

Superoxide dismutase (SOD) holds a pivotal role as the initial defense mechanism against ROS within cells, as highlighted by Alscher, Erturk and Heath^77^. This is attributed to the superoxide radical's role as a precursor to a range of highly reactive species. Maintaining a steady superoxide concentration through SOD regulation is vital for cellular protection, as emphasized by Fridovich^78^. Notably, SOD's activation is spurred by its own substrate, the superoxide radical itself^79,80^, indicating that heightened cellular SOD activity might signify stress induced by pollutant-triggered superoxide radicals. The impact of heavy metals, such as Cd and Cu, leads to a decrease in SOD activity due to metal ions binding to the enzyme's active center, as suggested by Bhaduri and Fulekar^81^. In higher plants, three major SOD isoforms exist: Cu,Zn-SOD in thylakoid membranes and the cytosol, Mn-SOD in mitochondria, and Fe-SOD in chloroplasts^82^. It's essential to note that the alterations in SOD observed in our study represent the total SOD activity, encompassing all isoforms. The decline in SOD activity is anticipated to result in increased H_2_O_2_ levels, as depicted in Fig. 2, D and A.

The accumulation of ROS induced by heavy metals, such as Cd and Cu, can potentially affect the activity of SOD enzymes. Cd and Cu toxicity are known to cause oxidative stress in plants by promoting ROS formation^83,84^. Exposure to low doses of Cd or Cu can increase SOD activity in plants, thereby protecting them from oxidative damage. However, high doses of Cd or Cu lead to a decrease in SOD activity^83^. Cd and Cu have a high affinity for sulfhydryl groups, which can result in metal-binding to these groups and alter antioxidant activity, including the activity of SOD enzymes, due to the inhibition of functional sulfhydryl groups^85^. Therefore, in this study, the decrease in SOD enzyme activity in the embryonic axes of zucchini seedlings may have been caused by the binding of sulfhydryl groups to Cd and Cu.

Glutathione S-transferase (GST) plays a pivotal role in the detoxification process of heavy metals and reactive oxygen species, while also contributing to redox balance regulation, as outlined by Siritantikorn, et al.^86^. This enzyme holds significant importance as a biomarker for heavy metal contamination^87^. Notably, our obtained results underscore a parallel pattern between changes in GST activity and MDA levels, indicative of lipid peroxidation. The heightened GST activity in response to metal-induced stress concurs with the findings of Labidi, et al.^57^ in zucchini, Jaouani, et al.^62^ in pea, and Hu, et al.^76^ in wheat, specifically in cases involving cadmium exposure.

Tau class glutathione S-transferase isoenzymes (GSTUs) have emerged as crucial defenders against metal-induced toxicity by orchestrating the conjugation of glutathione (GSH) with metal ions, leading to their sequestration within vacuoles, as noted by Dixon, Lapthorn and Edwards^88^ and Moons^89^. Kilili, et al.^90^ provided compelling evidence for the role of Tau class glutathione *S*-transferases (LeGSTUs) in orchestrating defense against oxidative stress. Through their adept regulation of the antioxidant response at the transcriptional level, these enzymes combat stress conditions, thwarting induced cell death, and safeguarding cell membranes from lipid oxidation initiated by H_2_O_2_ radicals. In the context of *Triticum* species breeding programs, Edwards, Dixon and Walbot^91^ revealed a robust correlation between heightened GST expression, resilience to environmental stressors, and augmented wheat yield. Roxas, et al.^92^ fortified this insight by demonstrating that elevated BI-GST enzyme expression, a Tau class homolog, bolsters resistance to salt and cold stress in tobacco seedlings. Furthermore, the study by Halušková, et al.^93^ illuminated the heightened GST activity in barley subjected to an array of heavy metals (Cd, Pb, Cu, Hg, Co, and Zn). In contrast, the work of Zhang and Ge^94^ highlighted the inhibition of specific GST isoenzymes in cadmium-exposed rice seedlings, in contrast to their active state in untreated counterparts. Drawing from these collective findings and the outcomes elucidated by Benhamdi, et al.^95^, it becomes evident that certain GST isoenzymes experience reduced functionality under metallic stress, while their Tau class counterparts undergo selective upregulation, substantiating the surge in GST activity, particularly notable in the context of 200 µM CdCl_2_ exposure within our study.

To assess the plant cell's capacity to mitigate H_2_O_2_, we evaluated the content of GSH, a pivotal constituent within plant cells, as underscored by Asgher, et al.^96^. Our investigations reveal that zucchini seedlings subjected to heavy metal treatments, such as Cd and Cu, exhibited a reduction in cellular GSH levels, as illustrated in Fig. 2F. These outcomes are in line with previous research that demonstrates how heavy metals like Hg, Cd, and Pb can perturb the cellular redox balance by depleting the GSH pool, as expounded by Okamoto, et al.^97^. The observed diminution of GSH in our study could be attributed to its potential utilization in the synthesis of phytochelatins, a mechanism proposed by Yadav^98^. Additionally, GSH might be involved in the regeneration of reduced ascorbate through the GSH-ASC cycle, as detailed by Asada^99^. The attenuated activities of CAT and SOD enzymes could also contribute to the observed decline in GSH levels.

In the context of glutathione, our findings align with the research of Ducruix, et al.^100^, Nagalakshmi and Prasad^101^, and Gallego, Benavides and Tomaro^102^, who collectively demonstrated that the GSH content diminishes in response to stress elicited by diverse metal concentrations, encompassing Cd, Cu, Fe, Zn, and others. The observed decline in GSH levels within our study can be attributed to its participation in detoxifying ROS radicals and metal ions, or to the potential inhibition of glutathione synthetase (GS) activity engendered by the presence of metals, as indicated by Hossain, et al.^103^. Additionally, the expedited reduction of oxidized glutathione (GSSG) through the enzymatic action of glutathione reductase (GR) under metal-induced stress^18^ may further contribute to the depletion of GSH levels. This diminution of GSH levels directly impacts the redox potential of the GSH/GSSG pair, generating a redox signal within cells exposed to stress conditions, as elucidated by Nocito, et al.^104^.

The outcomes concerning phytochemical parameters, encompassing total polyphenol (TPC) and flavonoid content (TFC), as well as antioxidant activity (*I*(%)) within the embryonic axis tissues of zucchini seedlings, illuminate a significant and proportional augmentation under the influence of elevated copper concentration (200 µM) within the growth medium, in distinct contrast to the response observed with cadmium (Cd). This substantiates the observations of Kısa, et al.^105^ in maize (*Zea mays* L.) and Badiaa, Yssaad and Topcuoglu^106^ in root tissues of tomato plants (*Lycopersicum lycopersicum* L.) subjected to copper-induced stress. The authors of these studies reported that the escalated presence of phenolic metabolites in plant cells functions to bolster their resilience under conditions of stress. Kısa, et al.^105^ further illustrated that phenolic compounds serve as efficacious metal chelators and potent scavengers of ROS, concurrently inhibiting the enzymes accountable for ROS production. In addition, the accumulation of flavonoids within and outside cell membranes plays a pivotal role in upholding membrane integrity by intercepting the ingress of deleterious elements into the cells^107^.

Contrastingly, the reduction in total phenols and the decline in antioxidant activity witnessed within the embryonic axis tissues as a consequence of exposure to 200 µM cadmium (CdCl_2_) can be attributed, as elucidated by Kısa, et al.^105^, to a discernible decrease in the enzymatic activity of pivotal enzymes implicated in the biosynthesis of phenolic compounds. This correlation aligns seamlessly with the outcomes of Okem, et al.^108^ in their investigation involving *Drimia elata*, where elevated concentrations of cadmium (CdNO_3_) (10 mg/L) were found to induce substantial inhibition in the synthesis of secondary metabolites like phenols and flavonoids. Additionally, cadmium's impact extends to the initiation of oxidative stress and the subsequent generation of ROS, which transpires due to alterations in the cellular composition of both enzymatic and non-enzymatic antioxidants^109^. Furthermore, the deleterious influence of Cd^2+^ on the plant's cellular metabolic processes yields inhibitory repercussions on the establishment of cellular ultrastructures, as attested by the investigations of El Rasafi, et al.^110^.

These findings collectively suggest that the oxidative stress induced by copper and cadmium in zucchini seedlings is complex, involving the interplay of various enzymatic and non-enzymatic antioxidants, as well as phytochemical compounds like polyphenols and flavonoids. The contrasting responses of these antioxidants to copper and cadmium exposure highlight the diverse strategies employed by plants to cope with heavy metal stress, which may vary depending on the specific metal and its concentration.

## Conclusion

In conclusion, the presence of copper and cadmium as heavy metals in the environment underscores their potential to exert detrimental effects on the growth and development of plants, even when encountered at relatively low concentrations. The findings of this study highlight that the influence of cadmium (Cd) and copper (Cu) can manifest as harmful and unpredictable consequences on the germination and vigor of zucchini seeds (*Cucurbita pepo* L.). This, in turn, leads to a risk of reduced development of the embryonic axes in young seedlings. It is noteworthy that these adverse effects of heavy metals on seedlings are closely linked to a metabolic imbalance, resulting in a state of oxidative stress. This oxidative stress arises from an increased accumulation of free radical species and a disturbance in both enzymatic and non-enzymatic antioxidant activity, attributed to exposure to metal ions.

## Data Availability

All data generated or analyzed during this study are included in this published article.
